# Supplementary material for: Nuclear Magnetic Resonance Dynamics of LiTFSI–Pyrazole Eutectic Solvents
Source: Materials (Basel). 2025 Nov 14;18(22):5184. doi: 10.3390/ma18225184 (PMC12654013; doi:10.3390/ma18225184)
Supplement: Supplementary file 1 [file materials-18-05184-s001.zip › materials-3962025-supplementary.pdf]

Supplementary Information

Nuclear Magnetic Resonance Dynamics of LiTFSI–Pyrazole Eutectic Solvents

Emilia Pelegano-Titmuss [1], Muhammad Zulqarnain Arif [2], Giselle de Araujo Lima e Souza [1,*], Phillip Stallworth [1], Yong Zhang [3], Adam Imel [2,4], Thomas Zawodzinski [2] and Steven Greenbaum [1]

[1] Department of Physics and Astronomy, CUNY Hunter College, New York, NY 10065, USA
[2] Department of Chemical and Biomolecular Engineering, University of Tennessee, Knoxville, TN 37996, USA
[3] Department of Chemical and Biomolecular Engineering, University of Notre Dame, Notre Dame, IN 46556, USA
[4] University of Tennessee-Oak Ridge Innovation Institute, Oak Ridge, TN 37830, USA
* Correspondence: giselle.souza@hunter.cuny.edu

Calculation of Spin Density (N_i)

The spin density (N_i, in units of spins·m^{-3}) for each nucleus i was calculated from the experimental density of the eutectic mixture and the mole fraction of the individual components. For a binary mixture of LiTFSI and pyrazole at a given stoichiometric ratio (1:X), the total molecular concentration c_{tot} was first obtained from the experimental density (ϱ) and the molar mass of the mixture (M_{mix}):

$$c_{\mathrm{tot}} = \frac{\rho}{M_{\mathrm{mix}}}$$

where ρ is expressed in g/m^3 and M_{mix} in g/mol.

The molar concentration of each component was then determined as:

$$c_j = x_j\, c_{\mathrm{tot}}$$

with x_j the mole fraction of component *j*.

Finally, the spin density for nucleus *i* was obtained as:

$$N_i = N_A \sum_j c_j n_{i,j}$$

where N_A is Avogadro's number and $n_{i,j}$ is the number of spins of type *I* per molecule of component *j*. For the systems studied here, pyrazole contributes 4 protons per molecule, while LiTFSI contributes 6 fluorine nuclei per molecule.

The calculated values of N_H and N_F for the investigated LiTFSI:PYR (1:X) eutectic solvents are summarized in Table S1.

Experimental densities (ρ) were estimated using temperature-controlled baths. Mixtures of crushed ice with sodium chloride provided approximate temperatures of –11.5 °C, –9 °C, –6.5 °C, and 0 °C, while a benzene/dry ice bath provided approximately –6 °C. A known mass of each eutectic mixture was transferred into a graduated cylinder, and the apparent volume was recorded at room temperature and after equilibration (30 min) in each bath. The density at –10 °C was estimated by extrapolating the volume–temperature trend. Because this method yields approximate values, densities are reported with two decimal digits to reflect the appropriate level of accuracy.

Table S1. Calculated spin density N_j for LiTFSI:PYR(1:X) eutectic systems.

LiTFSI:PYR(1:X)	ϱ (g/cm³)	N_H (m⁻³)	N_F (m⁻³)
(1:2)	1.48	1.68E+28	1.26E+28
(1:3)	1.27	1.87E+28	9.34E+27
(1:4)	1.35	2.01E+28	7.55E+27
(1:5)	1.17	2.25E+28	6.24E+27

Self-Diffusion Coefficients, Viscosity and Conductivity Raw Data and Calculated Ionicity

Table S2. LiTFSI–Pyrazole self-diffusion coefficients.

LiTFSI:PYR(1:X)	D ^{1}H (m²/s)	D ^{19}F (m²/s)	D ^{7}Li (m²/s)
(1:2)	9.62E-13	1.76E-12	5.09E-13
(1:3)	4.28E-12	3.20E-12	3.59E-12
(1:4)	6.25E-12	5.38E-12	4.49E-12
(1:5)	1.07E-11	7.86E-12	6.69E-12

Table S3. Experimental viscosity, conductivity and estimated ionicity.

LiTFSI:PYR(1:X)	Viscosity (Pa·s)	Conductivity (mS/cm)	Ionicity
(1:2)	0.678	0.60	0.06
(1:3)	0.402	1.30	0.05
(1:4)	0.277	3.13	0.07
(1:5)	0.373	2.28	0.02

Determination of the Intermolecular and Intramolecular Distances by MD Simulations

Molecular dynamics (MD) simulations were carried out using the package LAMMPS[1]. For each solvent, the simulation box was built by placing the appropriate number (see Table S4) of LiTFSI salt and PYR molecules randomly in a cubic box using the package Packmol [2,3]. Each system was then equilibrated for 2 ns in the isothermal–isobaric (NPT) ensemble to determine the density, followed by a 10 ns production simulation in the canonical ensemble (NVT) with the atomic coordinates saved every ps. The Nosé–Hoover thermostat [4] and the extended Lagrangian approach [5] were applied to control the temperature and pressure, respectively. A time constant of 100 fs was used in both the thermostat and barostat. The pressure was fixed at one atmosphere in all constant pressure simulations with isotropic volume fluctuations, and the temperature was set to 298 K. A timestep of 1 fs was used in all simulations.

Li^+ parameters were taken from the Optimized Potentials for Liquid Simulations (OPLS) force field [6]. The general Amber force field (GAFF) [7] was used to describe $TFSI^-$ anion and PYR molecules. To derive partial atomic charges used with GAFF, electronic structure calculation was carried out on each isolated ion at the B3LYP/6-311++g(d,p) level using the package Gaussian[8]. The atomic charges were then derived based on the optimized structure by fitting the electrostatic potential surface obtained from this calculation using the restrained electrostatic potential (RESP) method [9]. To approximate the effect of charge transfer and polarizability in the bulk phase, the partial charges of Li^+ and $TFSI^-$ were scaled to ±0.8 e, respectively[10]. The long-range electrostatic interactions were calculated using the particle–particle–particle–mesh (PPPM) method[11] with a real space cutoff of 12 Å. The same cutoff was used for van der Waals interactions, and a tail correction [12] was applied.

Table S4. Composition of simulation boxes.

LiTFSI:PYR(1:X)	# of LiTFSI	# of PYR
(1:2)	300	600
(1:3)	250	750
(1:4)	200	800
(1:5)	175	875

To improve the quality of the fitting, the intramolecular H–H distance was fixed to the minimum possible value (2.3 Å), as obtained from molecular dynamics simulations. For the intermolecular contributions, the probable distances extracted from molecular dynamics and used as fitting parameters were $d_{H\text{–}H}$ = 3.2 Å; $d_{F\text{–}F}$ = 5.1 Å; $d_{H\text{–}F}$ = 2.9 Å.

Table S5. Frequency-dependent R_1 relaxation data for ^1H nucleus in LiTFSI:PYR(1:X) eutectic systems at 10 °C.

	^1H R_1 (s^{-1})			
ν (Hz)	LiTFSI:PYR(1:2)	LiTFSI:PYR(1:3)	LiTFSI:PYR(1:4)	LiTFSI:PYR(1:5)
5.00E+08	0.481	0.462	0.435	0.625
4.00E+08	0.632	0.563	0.553	0.780
9.00E+07	2.080			
1.50E+07	12.139	11.705	10.920	8.240
1.08E+07	18.130	13.757	12.766	9.171
7.80E+06	24.291	15.860	14.400	9.726
5.62E+06	34.916	17.525	14.904	10.333
4.05E+06	48.022	19.855	16.161	10.574
2.92E+06	66.263	20.393	16.813	11.144
2.11E+06	87.219	21.354	17.532	11.303
1.52E+06	110.700	21.561	18.167	11.828
1.10E+06	131.660	22.909	18.389	11.823
789900	160.920	23.355	19.112	12.178
569610	183.460	23.375	19.191	12.241
410420	202.950	24.029	19.694	12.477
296050	221.750	24.635	19.657	12.285
213380	234.200	24.835	19.883	12.356
154070	249.580	25.737	20.049	12.701
111210	258.440	24.660	20.181	12.924
80210	267.280	25.811	20.730	12.998
57670	272.430	25.616	20.751	12.868
41710	274.910	25.533	20.733	12.894
29970	278.000	27.025	21.192	13.247

Table S6. Frequency-dependent R_1 relaxation data for ^{19}F nucleus in LiTFSI:PYR(1:X) eutectic systems at 10 °C.

	^{19}F R_1 (s^{-1})			
ν (Hz)	LiTFSI:PYR(1:2)	LiTFSI:PYR(1:3)	LiTFSI:PYR(1:4)	LiTFSI:PYR(1:5)
4.71E+08	2.025	2.049	2.014	1.952
3.77E+08	1.868	1.835	1.808	1.736
8.4e+07	3.530			
1.41E+07	9.009	9.082	7.277	6.364
1.02E+07	13.428	10.629	8.122	6.955
7.34E+06	17.894	13.041	10.327	7.848

5.29E+06	25.616	14.539	11.539	8.545
3.82E+06	34.043	16.084	11.789	8.975
2.75E+06	45.778	17.979	12.669	9.785
1.98E+06	61.414	18.478	13.164	10.324
1.43E+06	79.060	20.268	13.715	10.461
1.03E+06	94.549	21.204	14.175	10.944
743488	107.550	22.215	14.463	11.343
536141	127.010	22.487	14.809	11.415
386305	144.370	23.025	15.659	11.756
278655	154.720	24.096	16.237	12.145
200842	164.670	24.394	15.351	12.327
145017	167.380	24.292	16.197	11.959
104676	180.430	24.316	16.781	12.247
75497.1	178.510	26.099	15.841	12.237
54281.5	192.880	24.539	16.949	12.880
39259.2	197.670	25.317	16.882	12.192
28209.1	200.440	26.461	17.023	12.699

Table S7. Frequency-dependent R_1 relaxation data for ^{7}Li nucleus in LiTFSI:PYR(1:X) eutectic systems at 10 °C.

**LiTFSI:PYR(1:2)**		**LiTFSI:PYR(1:3)**	
**ν (Hz)**	**^{7}Li R_1 (s^{-1})**	**ν (Hz)**	**^{7}Li R_1 (s^{-1})**
1.94E+08	2.750	1.94E+08	3.551
1.55E+08	3.770	1.55E+08	5.257
1.24E+07	80.171	1.24E+07	85.8824
9.72E+06	106.684	9.71E+06	109.694
6.84E+06	158.625	6.60E+06	141.521
5.83E+06	203.874	4.78E+06	170.348
4.78E+06	249.266	3.36E+06	209.58
3.36E+06	408.143	2.36E+06	220.348
		1.65E+06	230.549
		1.16E+06	224.509
		815290	241.045
		571920	226.971
		401250	228.915
		281980	231.67
		197580	218.097
		97500	228.932
		48080	228.016
		33660	225.156
		19380	236.688
		11640	225.426
**LiTFSI:PYR(1:4)**		**LiTFSI:PYR(1:5)**	
**ν (Hz)**	**^{7}Li R_1 (s^{-1})**	**ν (Hz)**	**^{7}Li R_1 (s^{-1})**
1.94E+08	3.441	1.94E+08	3.310
1.55E+08	5.042	1.55E+08	4.690
1.24E+07	66.660	1.24E+07	40.871
8.63E+06	77.806	9.33E+06	50.836
5.98E+06	106.820	6.61E+06	47.336
4.12E+06	105.750	3.50E+06	58.019
2.87E+06	113.900	1.86E+06	59.262

1.99E+06	127.320	987000	60.111
1.38E+06	128.350	525000	55.592
952000	125.660	277000	56.128
661000	129.670	147000	57.565
459000	111.870	78100	58.387
317000	121.220	41400	54.677
219000	127.060	22000	57.655
152000	118.280	11600	52.828
105000	117.830		
72900	125.440		
50500	111.860		
35000	122.990		
24200	124.340		
16900	110.060		
11600	117.170		

References

1. Plimpton, S. Fast Parallel Algorithms for Short-Range Molecular Dynamics. *J Comput Phys* **1995**, *117*, 1–19, doi:10.1006/jcph.1995.1039.
2. Martínez, J.M.; Martínez, L. Packing Optimization for Automated Generation of Complex System's Initial Configurations for Molecular Dynamics and Docking. *J Comput Chem* **2003**, *24*, 819–825, doi:10.1002/jcc.10216.
3. Martínez, L.; Andrade, R.; Birgin, E.G.; Martínez, J.M. Software News and Update Packmol: A Package for Building Initial Configurations for Molecular Dynamics Simulations. *J. Comput. Chem* **2009**, *30*, 2157–2164.
4. Hoover, W.G. Canonical Dynamics: Equilibrium Phase-Space Distributions. *Phys Rev A (Coll Park)* **1985**, *31*, 1695–1697.
5. Shinoda, W.; Shiga, M.; Mikami, M. Rapid Estimation of Elastic Constants by Molecular Dynamics Simulation under Constant Stress. *Phys Rev B* **2004**, *69*, 134103.
6. Jorgensen, W.L.; Maxwell, D.S.; Tirado-Rives, J. Development and Testing of the OPLS All-Atom Force Field on Conformational Energetics and Properties of Organic Liquids. *J Am Chem Soc* **1996**, *118*, 11225–11236.
7. Wang, J.; Wolf, R.M.; Caldwell, J.W.; Kollman, P.A.; Case, D.A. Development and Testing of a General Amber Force Field. *J Comput Chem* **2004**, *25*, 1157–1174, doi:10.1002/jcc.20035.
8. Frisch, M.J.; Trucks, G.W.; Schlegel, H.B.; Scuseria, G.E.; Robb, M.A.; Cheeseman, J.R.; Scalmani, G.; Barone, V.; Petersson, G.A.; Nakatsuji, H.; et al. Gaussian 16 Revision C.01 2016.
9. Bayly, C.I.; Cieplak, P.; Cornell, W.; Kollman, P.A. A Well-Behaved Electrostatic Potential Based Method Using Charge Restraints for Deriving Atomic Charges: The RESP Model. *J Phys Chem* **1993**, *97*, 10269–10280, doi:10.1021/j100142a004.
10. Zhang, Y.; Maginn, E.J. A Simple AIMD Approach to Derive Atomic Charges for Condensed Phase Simulation of Ionic Liquids. *J Phys Chem B* **2012**, *116*, 10036–10048, doi:10.1021/jp3037999.
11. Hockney, R.; Eastwood, J. *Computer Simulation Using Particles*; Adam Hilger, New York, 1989;
12. Sun, H. COMPASS: An Ab Initio Force-Field Optimized for Condensed-Phase Applications Overview with Details on Alkane and Benzene Compounds. *J Phys Chem B* **1998**, *102*, 7338–7364, doi:10.1021/jp980939v.
